# Little Brown Bats (*Myotis lucifugus*) Support the Binding of SARS-CoV-2 Spike and Are Likely Susceptible to SARS-CoV-2 Infection

**DOI:** 10.3390/v15051103

**Published:** 2023-04-30

**Authors:** Shubhada K. Chothe, Padmaja Jakka, Veda Sheersh Boorla, Santhamani Ramasamy, Abhinay Gontu, Ruth H. Nissly, Justin Brown, Gregory Turner, Brent J. Sewall, DeeAnn M. Reeder, Kenneth A. Field, Julie B. Engiles, Saranya Amirthalingam, Abirami Ravichandran, Lindsey LaBella, Meera Surendran Nair, Costas D. Maranas, Suresh V. Kuchipudi

**Affiliations:** 1 Animal Diagnostic Laboratory, Department of Veterinary and Biomedical Sciences, The Pennsylvania State University, University Park, PA 16802, USA; skc172@psu.edu (S.K.C.);; 2 Center for Infectious Disease Dynamics, Huck Institutes of the Life Sciences, The Pennsylvania State University, University Park, PA 16802, USA; 3 Department of Chemical Engineering, The Pennsylvania State University, University Park, PA 16802, USA; 4 Pennsylvania Game Commission, 2001 Elmerton Ave, Harrisburg, PA 17110, USA; 5 Department of Biology, Temple University, Philadelphia, PA 19122, USA; 6 Department of Biology, Bucknell University, Lewisburg, PA 17837, USA; 7 Departments of Pathobiology and Clinical Studies, New Bolton Center, School of Veterinary Medicine, University of Pennsylvania, Kennett Square, PA 19348, USA

**Keywords:** SARS-CoV-2, ACE2, little brown bat, coronaviruses

## Abstract

Severe acute respiratory syndrome coronavirus-2 (SARS-CoV-2), believed to have originated from a bat species, can infect a wide range of non-human hosts. Bats are known to harbor hundreds of coronaviruses capable of spillover into human populations. Recent studies have shown a significant variation in the susceptibility among bat species to SARS-CoV-2 infection. We show that little brown bats (LBB) express angiotensin-converting enzyme 2 receptor and the transmembrane serine protease 2, which are accessible to and support SARS-CoV-2 binding. All-atom molecular dynamics (MD) simulations revealed that LBB ACE2 formed strong electrostatic interactions with the RBD similar to human and cat ACE2 proteins. In summary, LBBs, a widely distributed North American bat species, could be at risk of SARS-CoV-2 infection and potentially serve as a natural reservoir. Finally, our framework, combining in vitro and in silico methods, is a useful tool to assess the SARS-CoV-2 susceptibility of bats and other animal species.

## 1. Introduction

Severe acute respiratory syndrome coronavirus-2 (SARS-CoV-2), the cause of the coronavirus disease 2019 (COVID-19) pandemic, is widely believed to have originated from bats. In addition to humans, SARS-CoV-2 has demonstrated the ability to infect a wide range of non-human hosts [1,2,3,4,5,6,7,8,9,10,11]. Bats, the mammals of the order *Chiroptera*, have well-established phylogenetic and co-evolutionary relationships with a broad array of zoonotic viruses, including rabies virus, influenza virus, Hendra and Nipah viruses, Marburg virus, and severe acute respiratory syndrome-related (SARSr) coronaviruses [12,13,14,15,16]. Various bat species have also been reported to be involved in the spillover of various closely related Sarbecoviruses, such as SARS-CoV [17], MERS-CoV [18,19,20], and PED-CoV [21], to other mammalian host species. However, there is still limited information regarding the involvement of different bat species in the origin and maintenance of the severe acute respiratory syndrome coronavirus-2 (SARS-CoV-2) in the wild.

Little brown bats (*Myotis lucifugus*) are widely distributed in North America, found across much of Alaska, Canada, and the continental United States. Once the most abundant bat species in North America, little brown bats (LBBs) have undergone massive population declines in parts of the continent. However, in areas not yet decimated by the fungal pathogen that causes white-nose syndrome, these bats remain relatively abundant [22]. A pre-COVID-19 pandemic report suggested that a third of LBBs were infected with several distinct clades of Alphacoronaviruses, which persisted at low levels in the intestine [23]. While other bat species, such as those in the genus *Rhinolophus,* have been shown to harbor SARSr-CoV viruses [16], there is no evidence so far reporting the detection of SARS-CoV-2 or SARSr-CoV virus/nucleic acid in LBBs. A recent SARS-CoV-2 experimental infection study in big brown bats (*Eptesicus fuscus*) showed no evidence of infection, viral excretion, transmission, or tissue pathology [24], suggesting that big brown bats may be relatively resistant to SARS-CoV-2. In contrast, Egyptian Rousette bats (*Rousettus aegyptiacus*) experimentally infected with SARS-CoV-2 developed a transient infection in the nasal cavity, trachea, lung, and lung-associated lymphatic tissue and, in one case, successfully transmitted the infection to a contact-control bat [25]. Further, a recent experimental infection study also found Mexican free-tailed bats (*Tadarida brasiliensis*) to be susceptible to SARS-CoV-2 infection [26]. Given the vast global diversity of bats (1456 species currently recognized) [27] and the diversity of CoVs within them, including SARSr-CoVs, studies of additional bat species, including “model” bat species such as the LBB, are warranted [28].

In coronaviruses, their host range, tissue tropism, and pathogenesis are mainly determined by the receptor recognition of the spike (S) protein [29]. SARS-CoV-2 uses human angiotensin-converting enzyme 2 (ACE2) as its functional receptor to bind, enter, and infect host cells [29]. In line with efforts to evaluate the host range of SARS-CoV-2, researchers have characterized the binding ability of SARS-CoV-2 receptor binding domain (RBD) on S1 protein and various ACE2 orthologs from a large number of species. Among the different species belonging to the 11 animal orders studied, cynomolgus monkey (*Macaca fascicularis*) and chicken were observed to be the of closest and farthest evolutionary distance relative to humans, respectively, regarding the amino acid sequence identities in ACE2 receptor [30,31]. Further, among the different closely related ACE2 orthologs, those from Primates, Lagomorpha, Pholidota, Perissodactyla, most Carnivora, and most artiodactyls are observed to have varied binding affinities with SARS-CoV-2 RBD experimentally. However, the ones from Rodentia, Insectivora, Afrotheria, and Galliformes exhibited minimal to undetectable SARS-CoV-2 RBD binding interactions [30,31].

Correspondingly, as a critical unknown in determining the susceptibility and the role of various bat species in the ecology and evolution of SARS-CoV-2, the question of whether a host expresses functional ACE2 receptors that are compatible and accessible for virus binding needs to be addressed. Although two in vitro studies have shown the binding capacity of the LBB-ACE2 to SARS-CoV-2 RBD [30,32], it remains unclear if the viral binding capacity holds true in LBB tissues in response to a natural infection. More importantly, the pattern and levels of ACE-2 expression in intact LBB target tissues and their accessibility for SARS-CoV-2 binding are unknown. Notably, the Chinese rufous horseshoe bat (*Rhinolophus sinicus*), an Old-World bat species with significant ties to SARSr-CoVs, has been shown to have no observable binding to RBD in both in vitro studies. However, intestinal organoids derived from the same species are fully susceptible to SARS-CoV-2 infection and sustain viral replication [33]. This suggests that in vitro observations may not necessarily translate to the in vivo host susceptibility. Owing to these observations and the lack of reliable methodology to assess the ability of bat species to support SARS-CoV-2 binding, we sought to investigate the accessibility and compatibility of LBB ACE2 receptors to SARS-CoV-2 binding using a combination of experimental and computational methods. In the current study, human and cat intestinal tissues were used as positive control tissues and chicken as a negative control to demonstrate ACE2 protein expression and SARS-CoV-2 binding.

## 2. Materials and Methods

### 2.1. Experimental Animal Tissues

Little brown bat respiratory and gastrointestinal tissues were collected by Pennsylvania Game Commission in 2016 as a part of a collaborative study with Temple University and Bucknell University (Pennsylvania Game Commission permit number: 33085). A total of ten adults and ten juvenile male little brown bat tissues were collected for the study from the state of Wisconsin and New York. The study (DMR-17) was approved by the Bucknell University Institutional Animal Care and Use Committee (IACUC number: DMR-17), and all methods were applied in accordance with relevant guidelines and regulations. Tissues were briefly fixed in 10% neutral buffered formalin prior to routine histoprocessing, paraffin embedding, and sectioning at 5µm thickness prior to hematoxylin and eosin (H&E) histochemical and immunohistochemical staining.

### 2.2. Immunohistochemistry

The paraffin-embedded 5µm thick tissue sections were deparaffinized in xylene and rehydrated in ethanol. Following 10 min presoaking of the rehydrated sections in Tris-buffered saline (TBS), sections were subjected to heat-mediated antigen retrieval. Briefly, the slides were placed in 10 mM Tris 1mM EDTA buffer and heated at 90 °C for 10 min, following a cool-down period of 15 min. The antigen retrieved sections were blocked at 4 °C overnight using inactivated goat serum (diluted 25 µL/mL TBS), followed by a TBS wash. ACE2 protein was stained using the primary Anti-ACE2 antibody (Abcam catalog #ab15348), and TMPRSS2 was stained using a recombinant Anti-TMPRSS2 antibody (Abcam catalog #109131) for 1 h.

Following three washes with TBS, sections were further incubated with a secondary Anti-Rabbit IgG H&L antibody (Alexa Fluor 647) (Abcam catalog #ab150075) for 35 min. The sections were washed three times with TBS and mounted in ProLongGold antifade mountant with nuclear stain 4′,6-diamino 2phenylindole, dihydro-chloride (DAPI). Negative controls were performed, omitting the primary antibody staining. Following 24 h of curing the sections at room temperature, the sections were imaged using Echo Revolve Fluorescent Microscope.

### 2.3. Production of SARS-CoV-2 Spike Pseudovirus

SARS-CoV-2 spike pseudovirus was produced using the third-generation lentiviral packaging plasmids as described previously [34]. The transfer plasmid encoding luciferase and ZsGreen (BEI Resources, catalog #NR-52516), helper plasmid encoding Gag/pol (BEI Resources, catalog #NR-52517), Tat (BEI Resources, catalog #NR-52518), Rev (BEI Resources catalog #NR-52519), and plasmid encoding spike of SARS-CoV-2 Wuhan (BEI Resources catalog #NR-52514) (Addgene, Watertown, MA, USA) were transfected in HEK 293T cells propagated in DMEM with 10% FBS. The pseudovirus-containing supernatants were collected after 48 h of transfection and filtered through 0.45 µm membrane filters, and aliquots were stored at −80 °C until further use. The infectivity titer of SARS-CoV-2 spike pseudovirus was determined using 293T cells overexpressing the human ACE2 receptor. Briefly, the 293T cells overexpressing human ACE2 were infected with 10-fold serial dilutions of pseudovirus in a 96-well white/clear bottom plate (Thermo Fisher Scientific, Waltham, MA, USA, catalog #165306). At 72 h post infection, the plates were equilibrated to room temperature, and 100µL of BrightGlo luciferase assay reagent (Promega, Madison, WI, USA, catalog #E2620) was added to each well. The luminescence was measured using Luminometer (BioTek Synergy HTX Multi-Mode Microplate Reader, Agilent, Santa Clara, CA, USA).

### 2.4. Preparation of Recombinant SARS-CoV-2/RBD Antigen

The recombinant SARS-CoV-2/RBD antigen was produced as detailed earlier [35] for an independent study [36]. Briefly, the plasmid pSL1510 containing SARS-CoV-2 Wuhan spike RBD (pCAGGS-RBD) was transfected in Expi293F cells using Expi293 Expression System (ThermoFisher Scientific catalog #14524) as per the manufacturer’s instructions. The resultant protein from the cell culture supernatant was dialyzed in PBS and snap-frozen for storage at −80 °C. The recombinant SARS-CoV-2/RBD antigen was detected using Anti-6X His tag antibody (Abcam catalog #5000).

### 2.5. Virus Binding Assay

Virus binding assay with SARS-CoV-2 pseudovirus was performed as previously described with other RNA viruses such as influenza [37]. Briefly, deparaffinized and antigen-retrieved tissue sections were incubated with SARS-CoV-2 Wuhan pseudovirus for 2 h at 37 °C. Tissues incubated with TBS served as negative controls (Figure S6). Following the virus incubation, the tissue sections were blocked overnight with inactivated goat serum. The sections were immunostained with primary anti-SARS-CoV-2 spike protein S1 monoclonal antibody (HL6) (ThermoFisher Scientific catalog #MA5-36247). Following 60 min of incubation with the primary antibody, sections were washed and incubated with a secondary Anti-Rabbit IgG H&L antibody (Alexa Fluor 647) (Abcam catalog #ab150075) for 35 min. The sections were washed three times with TBS and mounted in ProLongGold antifade mountant with DAPI. Following 24 h of curing the sections at room temperature, the sections were imaged using Echo Revolve Fluorescent Microscope.

### 2.6. Homology Modeling and Complex Structure Generation Using HADDOCK

ACE2 homology models of LBB and chicken were prepared in the SWISSMODEL [38] webserver using the templates with the highest sequence identities. The 6LZG [39] complex of human-ACE2-RBD was used to identify all polar contacts, and these contacts were specified as constraints for docking in HADDOCK [40] with the default protocol run on their webserver. The models with the least energy of the largest populated cluster were used for further binding energy and MD simulations.

### 2.7. Rosetta Simulations

Rosetta binding energy calculations were performed by first running the relax application with the ref2015 [41] energy function and extracting binding energy (dG_separated) using the InterfaceAnalyzer [42] application. Three hundred independent relax simulations were performed, and the top 10% output structures with the least Rosetta energy of complex were selected for computing the binding energy. Rosetta docking calculations were performed on the ROSIE2 online server [43].

### 2.8. Molecular Dynamics Simulations

Each protein complex was used to start four independent 100-nanosecond trajectories with random initial velocities. The ACE2-RBD (with and without RBD) complexes were prepared for molecular dynamics (MD) simulations using the protein preparation wizard of Maestro [44]. The hydrogen-bonding network was first optimized, and a heavy atom-restrained minimization with the OPLS3e [45] was carried out. Each complex was then solvated with water using the TIP3p [46] model in an orthorhombic box with 10-Å buffer distance in each dimension. The residual charges from the system were neutralized by adding Na^+^ and Cl ions to maintain a 0.15 M salt concentration. MD simulations were performed using the Desmond [47] application within the Schrodinger software suite (v2019.4). The default relaxation protocol of Desmond was performed, followed by 100 ns of production simulations at 1 atm pressure and 300 K temperature, using the NPT ensemble under a periodic boundary condition using particle mesh Ewald [48]. A Time step of 2.0 fs and a nonbonded cut-off threshold of 9-Å were imposed. Finally, the SHAKE [49] algorithm was used to keep all bonds involving hydrogen atoms rigid. All MD trajectory analyses were performed using the Schrodinger suite’s MD scripts.

### 2.9. Phylogenetic Analyses

The human, cat, chicken, little brown bat, big brown bat, and Mexican free-tailed bat ACE2 protein sequences were obtained from the GenBank database. The phylogenetic tree was constructed using Geneious prime 2022.1.1 software. The list of species and GenBank IDs is provided in Table S1.

## 3. Results

### 3.1. Differential Distribution of ACE2 Receptor in LBB Upper and Lower Respiratory Tract

**Figure 1:**
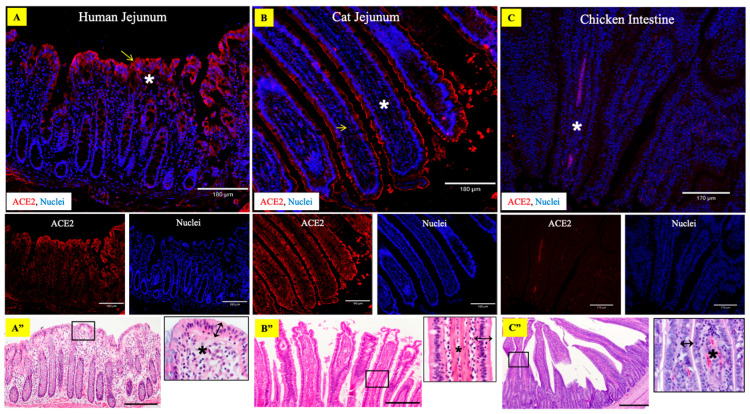
Expression of ACE2 protein receptors in human (**A**), cat (**B**), and chicken (**C**) intestines. Composite fluorescent images of human and cat jejunum showed abundant expression of ACE2 receptors within simple columnar epithelial cells of the intestinal villi (arrows) with rare cells in the lamina propria (asterisk), whereas the chicken intestine has low ACE2 expression scattered throughout the lamina propria only (asterisk). The tissue sections were stained with rabbit polyclonal antibody to ACE2 (red) and DAPI nuclear stain (blue). (**A’’**): hematoxylin and eosin (H&E) stained human intestine tissue section with high magnification inset of the framed area highlighting simple columnar epithelium (double-headed arrows) and lamina propria (asterisk). Scale bar = 180 µm. (**B”**): H&E stained cat intestine tissue section with high magnification inset of the framed area highlighting simple columnar epithelium (double-headed arrows) and lamina propria (asterisk). Scale bar = 180 µm. (**C”**): H&E stained chicken intestine tissue section with high magnification inset of the framed area highlighting simple columnar epithelium (double-headed arrows) and lamina propria (asterisk). Scale bar = 180 µm.

**Figure 2:**
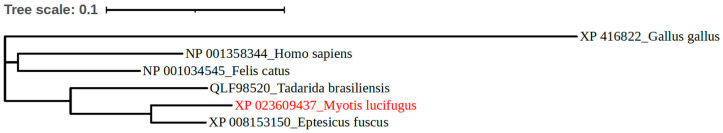
Phylogenetic tree based on ACE2 protein sequences for selected species. The tree was constructed using the complete ACE2 protein sequences of North America’s three most common bat species, Little brown bats (*Myotis lucifugus*), Big brown bats (*Eptesicus fuscus*), Mexican free-tailed bats (*Tadarida brasiliensis*), and other animal species used in this study (cat, chicken, and human). The Human ACE2 had higher sequence similarities with all the species except for chicken.

Abundant tissue distribution of ACE2 protein in the human and cat gastrointestinal tract (GIT) has been described previously [31,50,51]. These studies have demonstrated a higher expression of ACE2 receptors in the small intestine than in the lungs. In contrast, it has been suggested that the chicken ACE2 protein structure varies significantly from other domestic animals and primates and hence may not interact with SARS-CoV-2; consequently, this virus may not infect them [52]. Our results were consistent with these observations (Figure 1). Further, a phylogenetic analysis based on ACE2 protein sequences demonstrated close to 80% similarity in the human and bat ACE2 versus only 65% similarity in the human and chicken ACE2 (Figure 2 and Figure S1). Based on the ACE2 receptor distribution studies and the computational analysis we performed, human and cat intestine tissues were selected to use as positive controls and chicken intestinal tissue as a negative control to validate our ACE2 immunohistochemistry staining in LBB gastrointestinal and respiratory tract.

**Figure 3:**
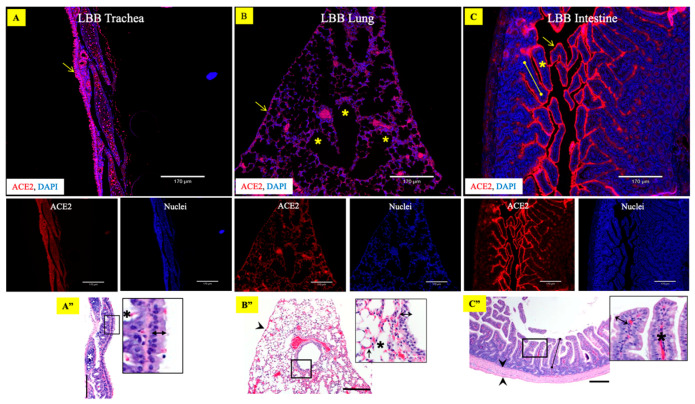
Expression of ACE2 protein receptors in little brown bat’s respiratory and gastrointestinal tract. Composite fluorescent images show expression of ACE2 protein receptor (red) on the (**A**) trachea, (**B**) lung, and (**C**) intestine. The ACE2 receptor was predominantly expressed on the mucosal epithelium of the trachea ((**A**), arrowhead), whereas it was moderately expressed in the lung tissue (**B**), including visceral pleura (arrow) and epithelium lining airways (asterisks). The ACE2 receptor in the intestine (**C**) predominated in the epithelial lining (arrows) villi (capped line), not the lamina propria (asterisk). The tissue sections were stained with rabbit polyclonal antibody to ACE2 (red) and DAPI nuclear stain (blue). Scale bar = 170 µm. (**A’’**): Hematoxylin and eosin (H&E)-stained tracheal tissue section with high-magnification inset of the framed area including respiratory epithelium (double-headed arrow) and submucosal glands (black asterisk), supported by hyaline cartilaginous rings (white asterisk). Scale bar = 170 µm. (**B”**): H&E-stained lung tissue section with high-magnification inset of the framed area including visceral pleura (arrowhead), the bronchiolar respiratory epithelium (double-headed arrows), alveolar duct (asterisk), alveolar blood capillary (arrow). Scale bar = 170 µm. (**C”**): H&E-stained intestinal tissue section with high-magnification inset of the framed area including villus (capped line), muscularis (arrowheads), simple columnar epithelial cell layer (double-headed arrows), and lamina propria (asterisk). Scale bar = 170 µm.

Owing to ~80% homology observed between LBB and human ACE2 proteins, the human anti-ACE2 antibody was used for the immunohistology staining. The relative abundance of ACE2 receptors was distinctly varied in the upper and lower respiratory tract of the LBB. While the ACE2-specific immunohistochemistry showed robust expression of ACE2 on the mucosal lining of the LBB trachea (Figure 3A), the alveolar epithelium, alveolar duct, and visceral pleura of the lung revealed a relatively lower distribution of the ACE2 receptors (Figure 3B). The lamina propria and submucosa of the tracheal tissue also showed a lesser distribution of the receptors. A total of ten adult and ten juvenile LBB respiratory tissues were investigated alongside the aforementioned positive and negative controls (Figure S2). No observable difference was noticed in the respiratory tract ACE2 distribution of adult and juvenile LBBs.

### 3.2. Abundant Expression of ACE2 Receptors in the LBB GIT

The small intestinal tissue sections of LBB, subjected to ACE2-specific immunohistochemistry using the human anti-ACE2 antibody, showed abundant expression of the ACE2 receptor on the intestinal epithelial cells (Figure 3C). The ACE2 immunolabeling was more prominent in epithelial cells distributed along the apical villi. Whereas the immunolabeling in other tissues of the small intestine was sporadic and low relative to the villus epithelium, including the crypt cells, lamina propria, muscularis, and intestine serosa. Moderate ACE2 was expressed by the secretory cells of the submucosal (Brunner’s) glands in the duodenum.

### 3.3. Abundant Expression of TMPRSS2 in LBB Trachea and GIT

Similar to ACE2, previous studies have also shown that a plasma membrane-associated type II transmembrane serine protease (TMPRSS2) promotes SARS-CoV-2 infection [53,54]. TMPRSS2 was also found to be abundantly expressed in the LBB trachea and small intestine of LBB and moderate in the lungs (Figure S3).

### 3.4. ACE2 Receptors in the LBB Trachea and GIT Support SARS-CoV-2 Binding

**Figure 4:**
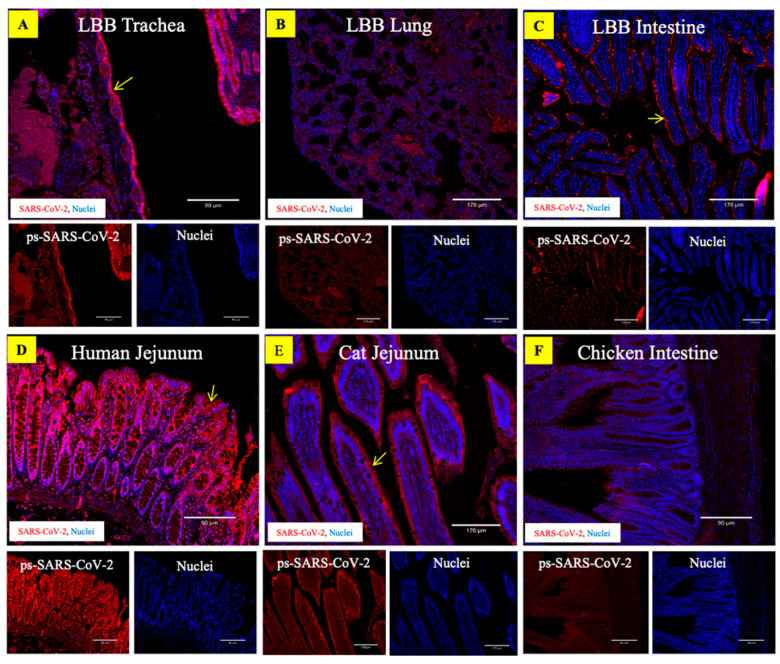
Binding of pseudo-SARS-CoV-2 virus to little brown bat (LBB) tissues. The composite fluorescent images show abundant binding of the pseudo-SARS-CoV-2 virus to the mucosal lining of the LBB trachea (**A**) and intestine (**C**). The LBB lung tissue (**B**) showed low-to-moderate binding of the virus. Human and cat jejunum (**D**,**E**), positive control tissues, showed preferential binding of the virus to the intestinal epithelium; however, chicken intestine (**F**), the negative control tissue exhibited minimal virus binding. The tissue sections were stained with rabbit anti-SARS-CoV-2 spike protein S1 monoclonal antibody (red) and DAPI nuclear stain (blue). Scale bar = 170 µm.

**Figure 5:**
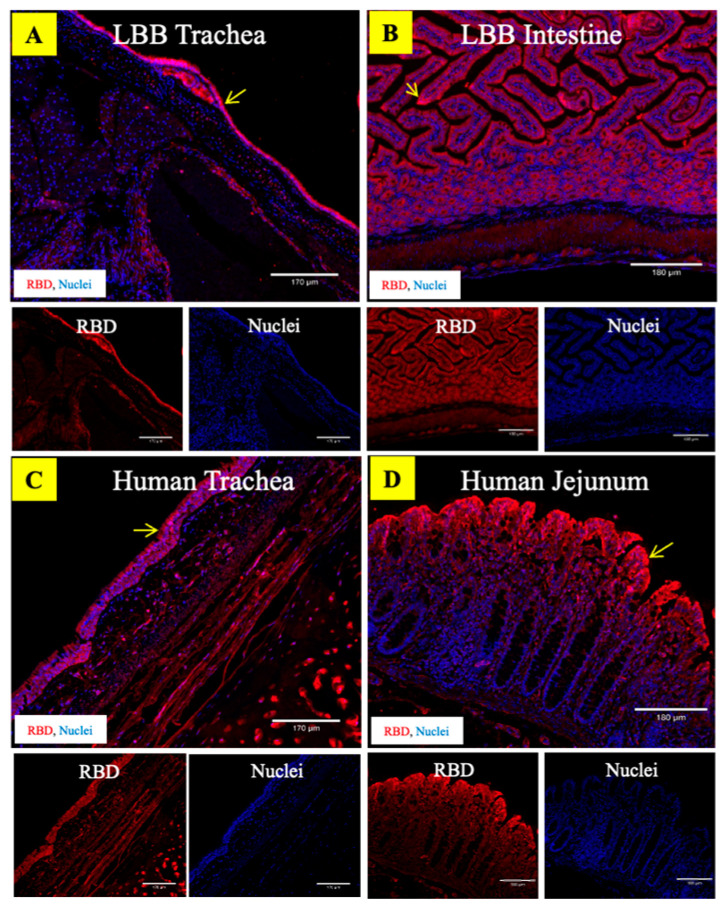
Binding of recombinant SARS-CoV-2/RBD antigen to the trachea and intestine of LBB and humans. The composite fluorescent tissue images show the binding of the recombinant SARS-CoV-2/RBD antigen to the ACE2 receptors on LBB trachea (**A**), LBB intestine (**B**), human trachea (**C**), and human jejunum (**D**). The recombinant SARS-CoV-2/RBD antigen is preferentially bound to the epithelial cells on the mucosal lining of the trachea and the intestine in both LBB and human tissues. The tissue sections were stained with rabbit polyclonal antibody to spike protein (red) and DAPI nuclear stain (blue). Scale bar = 170 µm.

We used two alternative approaches to demonstrate the binding interactions between the ACE2 receptor and the SARS-CoV-2 in LBB tissues. In the first approach, virus–receptor binding assays were performed on LBB trachea and intestine tissue as well as the human, cat, and chicken intestine tissue using the SARS-CoV-2 spike pseudovirus (ps-SARS-CoV-2) generated in our laboratory. The LBB ACE2 receptors in the trachea and intestine were compatible with binding to the ps-SARS-CoV-2 (Figure 4A–C). The virus binding pattern was consistent with the relative abundance of the ACE2 receptors, such that greater ACE2 expression correlated with more significant virus binding. The controls, human and cat intestinal tissue, also demonstrated high and moderate levels of ps-SARS-CoV-2 binding, respectively, to the ACE2 receptors compared to the negative control chicken intestinal tissue (Figure 4D–F). The mock-treated tissues (Figure S4) exhibited minimal background staining. In the second approach, trachea and intestinal tissue from bats and humans were also subjected to binding with recombinant SARS-CoV-2/RBD antigen that was produced as described previously [35]. The RBD binding pattern also followed the relative distribution of ACE2 in these tissues (Figure 5), validating the potential ability of LBB ACE2 to function as an entry receptor for the virus. The negative control tissue for the recombinant SARS-CoV-2/RBD antigen-binding assay showed minimal background staining as no secondary antibody was used in the assay (Figure S5).

### 3.5. Molecular Simulations Reveal Compatibility of SARS-CoV-2-Spike Receptor Binding Domain with LBB

**Figure 6:**
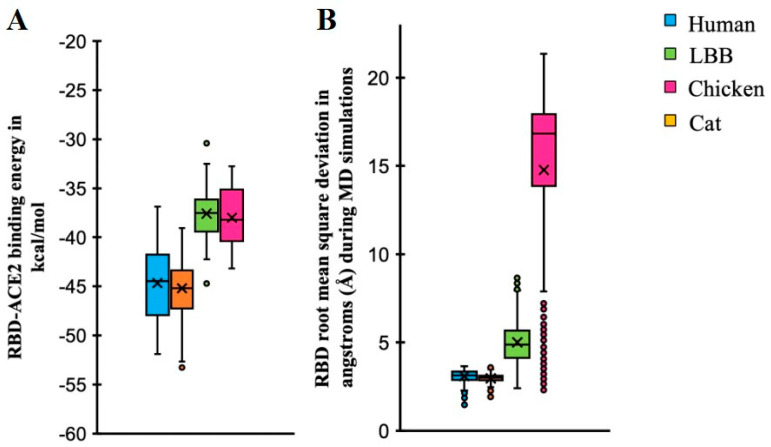
Binding energy simulation results for ACE2-RBD complexes. (**A**) Binding energy simulation results for ACE2-RBD complexes for species under investigation indicate that the mean binding energies of human and cat ACE2s with RBD are ~8 kcal/mol stronger compared to those of LBB and Chicken ACE2s. (**B**) Root mean square deviation of RBD during 400 ns MD simulations of ACE2-RBD complexes. The ACE2-RBD complexes of species under investigation are simulated, and RBD deviation at each time point is measured with respect to its initial coordinates after superposing the ACE2 coordinates. RBD deviates the most (by 16.8 Å) when bound to chicken-ACE2 compared with others.

**Figure 7:**
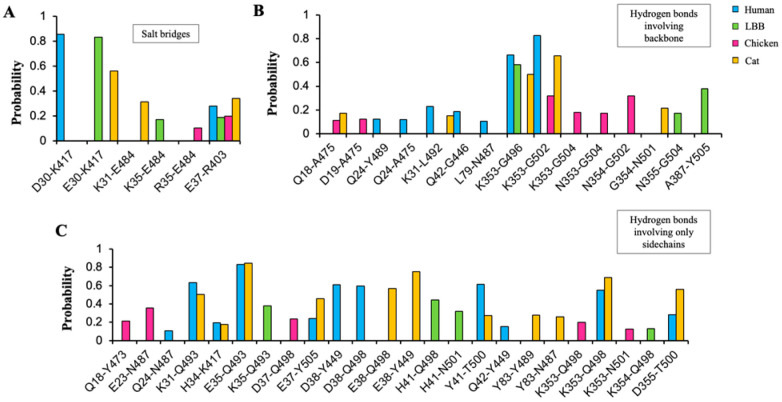
ACE2-RBD interactions in human, LBB, chicken, and cat. Bar plots showing the probabilities of (**A**) salt-bridge interactions, (**B**) hydrogen bonds involving backbone atoms, and (**C**) hydrogen bonds involving only sidechain atoms. The number of frames each interaction formed at the ACE2-RBD interfaces during 400 ns MD simulations is counted and is used to calculate the respective probability as the fraction of frames the interaction is present. The numbering of residues follows the PDB structures 6LZG and 7C8D for the Human ACE2-RBD and cat ACE2-RBD complexes. For LBB and chicken, the numbering follows the aligned sequences as displayed in Figure S1.

The three-dimensional structures of the ACE2-RBD complexes for LBB and chicken were prepared using homology modeling [38] and docking refinement using HADDOCK [40] (see methods). The modeled docked complexes were validated for the accuracy of the binding location as described previously [55] based on whether RosettaDock [43] (see methods) produced docking funnels (Figures S7 and S8). The Rosetta binding energies were calculated to predict the strength of binding affinity of ACE2 from different species to SARS-CoV-2 spike RBD (see methods). The mean binding energies of human ACE2-RBD and cat ACE2-RBD complexes were significantly more negative than those of LBB ACE2 and chicken ACE2 (Figure 6A), indicating comparatively stronger affinities of human and cat ACE2s (‘higher’ binding energy implies lower affinity and vice-versa). This observation is congruent with the in vitro studies that showed that the LBB ACE2 had approximately 15 times weaker binding affinity compared to that of human ACE2 [30]. However, the binding energy alone does not explain why chicken ACE2 had similar binding energy yet had negative binding in experiments (Figure 6A). To investigate this further, we performed all-atom molecular dynamics (MD) simulations for all the respective protein complexes (human-ACE2, cat-ACE2, LBB-ACE2, and chicken-ACE2). Four independent replicate simulations starting from different random initial velocities for atoms were performed, yielding a 400 ns simulation trajectory for each complex. All replicate simulation results showed that ACE2 from human, cat, and LBB form stable complexes, while the chicken ACE2 protein shows an unstable complex with RBD. This was further quantified by calculating the root mean square deviation of RBD coordinates (keeping ACE2 coordinates fixed) from its initial position during the simulations (Figure 6B). These results suggest that the LBB-ACE2 is predicted to form a stable complex with RBD, which is comparatively weaker than human and cat-ACE2 proteins but is likely strong enough to initiate infection. While the chicken-ACE2 was predicted to have similar binding energy to LBB-ACE2 (Figure 6A), it did not form a stable complex, suggesting a lack of binding to RBD (Figure 7), which is consistent with experimental data [30,32]. Having confirmed the formation of a stable LBB-ACE2 and RBD complex, we further characterized the functional interactions at the protein–protein interface.

**Figure 8:**
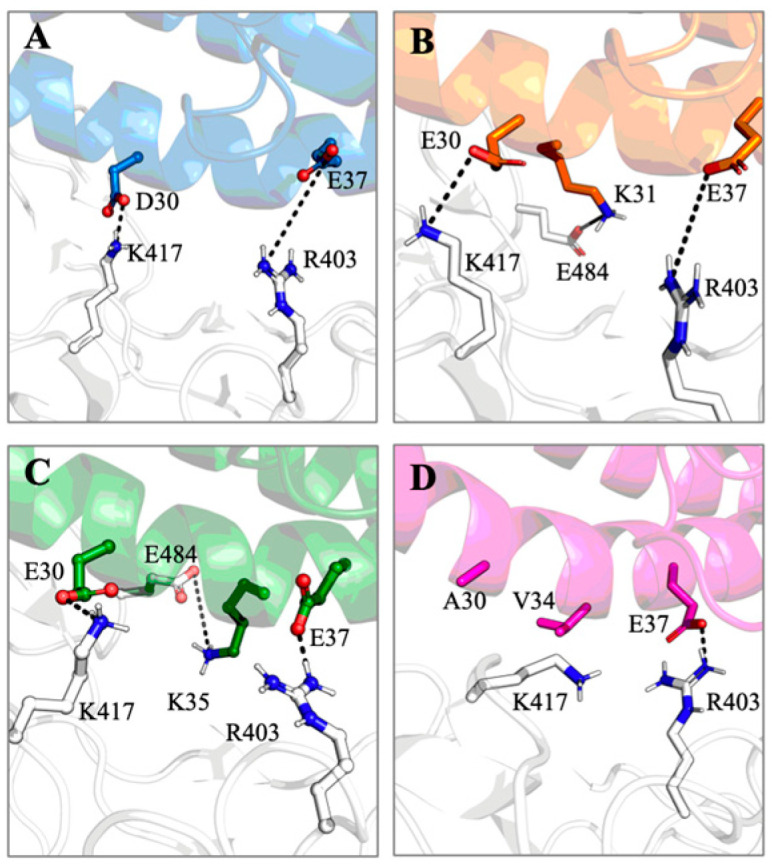
Salt-bridge interactions formed at the ACE2-RBD interface of various species. (**A**) human–ACE2, (**B**) cat–ACE2, (**C**) LBB–ACE2, and (**D**) chicken–ACE2. Chicken–ACE2 lacks the necessary residues in proximity to form salt bridges compared to others except with the E37-R403 residue.

The human and cat-ACE2 proteins formed 20 and 19 interactions involving salt bridges and hydrogen bonds, respectively, while the LBB and chicken ACE2s formed 10 and 13 interactions, respectively (Figure 7). Interactions that were seen in less than 10% (<0.1 probability) of the frames during the simulations were not counted. The human and cat-ACE2s had ~70% common interactions with the RBD (Figure 7), also corroborating their similar binding energies. Interestingly, the strongest electrostatic interactions D30-K417 formed at the human-ACE2-RBD interface were also formed by cat-ACE2 and LBB-ACE2 as E30-K417 but not by chicken ACE2 (Figure 8), which has hydrophobic residues at the corresponding positions. Similar to cat-ACE2, LBB-ACE2 also forms a salt-bridge interaction with the E484 residue of the RBD (Figure 8B,C). Strong electrostatic interactions such as salt bridges provide dominant long-range forces and guide functional protein-protein association [56]. These results suggest that the lack of strong electrostatic interactions render the chicken ACE2 unable to bind to RBD by destabilizing the complex.

## 4. Discussion

Bats serve as reservoirs of hundreds of coronaviruses capable of spillover into human populations. Previous studies reported the presence of SARSr-CoVs in various Old-World bat species, including horseshoe bats in the genus *Rhinolophus* [57]. SARS-CoV-2 is known to infect a wide range of non-human animal hosts. While it is shown that SARS-CoV2 uses ACE2 for entry and TMPRSS2 for S protein priming, other host factors such as neuropilin-1 and receptor tyrosine kinase AXL are also found to facilitate viral entry [54,58,59]. Further, proteases such as cathepsin L and cathepsin B can play an important role in coronavirus entry by cleaving and activating spike protein for the entry [60]. In vitro experimental studies using human cells expressing bat ACE2 orthologues suggest a significant variation in susceptibility among bat species to SARS-CoV-2 infection [61]. Consequently, recent experimental studies show that big brown bats (*Eptesicus fuscus*) are resistant to SARS-CoV-2 infection. In contrast, Mexican free-tailed bats (*Tadarida brasiliensis*) [26] and Egyptian Rousette bats (*Rousettus aegyptiacus*) [25] are found to be susceptible to experimental SARS-CoV-2 infection. However, the susceptibility of several North American bat species to SARS-CoV-2 infection has yet to be fully understood. A key determinant of host susceptibility is the expression of ACE2 receptors on target tissues that are compatible and accessible for virus binding. Using immunohistochemistry, virus binding assays, Rosetta binding energy calculations, and all-atom molecular dynamics (MD) simulations, we show that LBB, a widely distributed bat species in North America, express ACE2 in their respiratory and GI tracts that support SARS-CoV-2 binding and therefore, could be susceptible to SARS-CoV-2 infection.

We found that LBBs express abundant levels of the ACE2 receptors in their upper airway as well as small intestinal villus epithelium and can functionally bind SARS-CoV-2, indicating the possibility that, should LBBs be exposed to the virus, they could become infected. This raises the possibility that if the infection did occur in wild LBBs, these bats could contribute to the transmission of the virus in North America. The tracheal epithelium in LBB showed prominent expression of ACE2 protein and was observed to bind to both ps-SARS-CoV-2 and recombinant SARS-CoV-2/RBD antigen. Nevertheless, the lower respiratory tract, particularly the lung tissue, only had a low-to-moderate expression of the ACE2 receptors.

With regards to ACE2 protein localization, various human tissues, such as the nasopharynx, lung, stomach, and small intestine, have demonstrated prominent receptor expression [50]. Similarly, ACE2 protein expression has been demonstrated in the gastrointestinal tract of domestic cats [31], whereas independent experimental studies have shown that species such as chickens and ducks are not susceptible to SARS-CoV-2 infection [62]. Based on this evidence, in this study, human and cat intestinal tissues were used as positive control tissues to demonstrate ACE2 protein expression and SARS-CoV-2 binding. In contrast, chicken intestinal tissue was utilized as a negative control.

Molecular simulations are being extensively used to model the ACE2-RBD interactions to predict the susceptibilities of different species to the wild-type SARS-CoV-2 and its different variants [63,64]. Such studies are valuable for large-scale surveillance studies to draw inferences on zoonotic origins [65], host susceptibilities [66], and viral adaptabilities [67]. In this study, we used molecular simulations to predict the binding affinity and the interactions involved in binding the LBB-ACE2 protein with the SARS-CoV-2 RBD. Based on previous observations and reports, corresponding known simulations of the human-ACE2 and cat-ACE2 complexes with RBD were used as positive controls, and those of the chicken ACE2-RBD modeled complex structure were used as negative controls for comparison.

Previously, it has been shown that a kidney cell line derived from the big brown bat [68] and lung cell lines derived from Lander’s horseshoe bat (*Rhinolophus landeri*) and Daubenton’s bat (*Myotis daubentonii*) could not be infected with SARS-CoV-2 [54]. Further, an experimental challenge of big brown bats with SARS-CoV-2 via nasal and oropharyngeal route showed that these bats are resistant to infection with the virus [24]. This could be due to either the overall low ACE2 expression in the respiratory tract or the presence of structurally different ACE2 protein which limits the binding to SARS-CoV-2 [69]. Considering the distinct bat species studied in this regard, the previous observations cannot be directly extended to LBBs or other bat species; nonetheless, our study indicates that there is only a minimal expression of ACE2 in LBB bat lungs and that the virus binding was not as prominent in the lung tissues when compared to that in the upper respiratory tract or parts of the intestines.

Additionally, similar to humans, the ACE2 protein was highly expressed in intestinal epithelial cells lining the villi in LBB and observed to bind to SARS-CoV-2, indicating the functional competence of LBB ACE2 as a SARS-CoV-2 receptor to promote entry into the LBB GIT. In line with our observations, infection of bat intestinal organoids by SARS-CoV-2 has also been demonstrated recently in an organoid culture of Chinese rufous horseshoe bats (*Rhinolophus sinicus*) [33]. Considering the lack of complete information regarding the ecological, behavioral, and genetic diversity of the bat species under the order *Chiroptera*, extrapolation of any results needs to be cautiously executed since observations made in one bat species may not hold true for others. Therefore, the potential SARS-CoV-2 infection susceptibility of other North American and, indeed, global bat species in addition to LBBs remains largely unknown. Our limited understanding of the role of bats in SARS-CoV-2 biology raises a critical need for comprehensive surveillance of different bat species for their role in maintaining SARSr-CoVs in the wild.

SARS-CoV-2 continues to evolve and the emerging variants could have altered host tropism and potentially gain the ability to infect new host species. For example, the mouse ACE2 did not bind to the original SARS-CoV-2 spike protein but was bound with higher affinity to spike proteins of the BA.1 and BA.2 variants [47]. Experimentally determined structures of mouse ACE2 bound to BA.1 RBD revealed an interaction network similar to that seen in the human ACE2-RBD complex [70]. Both molecular dynamics-based and force-field-based methods are well suited for simulating the interactions of protein complexes and predicting mutations in proteins responsible for increasing or decreasing binding affinities [71]. However, such methods are less accurate for predicting actual protein-protein binding affinities [72]. Recently emerging machine-learning algorithms are combining structure and force-field calculations to achieve better accuracies at reproducing experimental binding affinities [73,74]. Such methods rely on the presence of high-throughput mutational datasets, which are currently limited to human protein complexes [74,75]. The development of computational surveillance methods focused on animal species is still lacking and is crucial to detect potential spillovers to combat current and emerging pandemics such as COVID-19.

In conclusion, we observed prominent expression of ACE2 in the LBB respiratory and gastrointestinal tract that are accessible for SARS-CoV-2 viruses to bind. We also observed a stable complex formation of LBB-ACE2 with the SARS-CoV-2 RBD but with a weaker binding affinity when compared to the cat- and human-ACE2 proteins in silico. Our results indicate that LBB, a widely distributed North American bat species, could be at risk of SARS-CoV-2 spillover infection and potentially serve as a natural reservoir and warrant targeted surveillance of LBB. Given the wide variety of bats (1456 species), it is not practically feasible to perform experimental infections to determine their susceptibility to SARS-CoV-2. Therefore, our study provides a practical framework comprising a combination of in vitro and in silico methods to establish the ability of a given animal species to support SARS-CoV-2 binding. Further investigation can be performed to determine the susceptibility of LBB species to SARS-CoV-2 infection using LBB respiratory and gastrointestinal cells. These experiments will also allow a detailed understanding of the disease pathology in this species. The accuracy of molecular simulation models can further be improved by incorporating data from new experiments to validate predictions. Continued use of molecular simulations will allow the prediction of the susceptibility of different species to SARS-CoV-2 and its variants.

## 5. Conclusions

Our study utilized a molecular simulation model to predict the binding affinity and interactions involved in LBB ACE2 and SARS-CoV-2 RBD. Further, these observations were validated with experimental studies showing that LBBs express ACE2 in their respiratory and gastrointestinal tracts, indicating their susceptibility to SARS-CoV-2 infection. Additional in vitro and in vivo studies are warranted to determine the susceptibility of other North American and global bat species to SARS-CoV-2 infection, as well as the role of little brown bats in the virus transmission.

## Data Availability

Data is contained within the article or supplementary material.

## Supplementary Materials

The following supporting information can be downloaded at: https://www.mdpi.com/article/10.3390/v15051103/s1, Figure S1: Sequence alignment of ACE2 proteins of human, cat, LBB, and chicken, Figure S2: ACE2 primary antibody mock staining, Figure S3: Expression of TMPRSS2 protein receptors in the trachea and intestine of the little brown bat, Figure S4: Mock staining for ps-SARS-CoV-2 virus binding assay, Figure S5: Mock staining for recombinant SARS-CoV-2/RBD antigen-binding assay, Figure S6: Mock staining of human tissues for SARS-CoV-2 spike pseudovirus binding assay, Figure S7: Molecular dynamics simulation trajectories showing RBD deviation during four independent 100ns replicate simulations, and Figure S8: Docking funnels for Rosetta docking simulations for ACE2-RBD complexes, Table S1: Species of interest and ACE2 GenBank identification number used in phylogenetic analyses.

## References

[1] Haagmans B.L., Koopmans M.P.G. (2022). Spreading of SARS-CoV-2 from hamsters to humans. Lancet.

[2] Gortazar C., Barroso-Arevalo S., Ferreras-Colino E., Isla J., de la Fuente G., Rivera B., Dominguez L., de la Fuente J., Sanchez-Vizcaino J.M. (2021). Natural SARS-CoV-2 Infection in Kept Ferrets, Spain. Emerg. Infect. Dis..

[3] Oreshkova N., Molenaar R.J., Vreman S., Harders F., Oude Munnink B.B., Hakze-van der Honing R.W., Gerhards N., Tolsma P., Bouwstra R., Sikkema R.S. (2020). SARS-CoV-2 infection in farmed minks, the Netherlands, April and May 2020. Euro Surveill.

[4] Chaintoutis S.C., Thomou Z., Mouchtaropoulou E., Tsiolas G., Chassalevris T., Stylianaki I., Lagou M., Michailidou S., Moutou E., Koenen J.J.H. (2021). Outbreaks of SARS-CoV-2 in naturally infected mink farms: Impact, transmission dynamics, genetic patterns, and environmental contamination. PLoS Pathog..

[5] Sit T.H.C., Brackman C.J., Ip S.M., Tam K.W.S., Law P.Y.T., To E.M.W., Yu V.Y.T., Sims L.D., Tsang D.N.C., Chu D.K.W. (2020). Infection of dogs with SARS-CoV-2. Nature.

[6] Mohebali M., Hassanpour G., Zainali M., Gouya M.M., Khayatzadeh S., Parsaei M., Sarafraz N., Hassanzadeh M., Azarm A., Salehi-Vaziri M. (2022). SARS-CoV-2 in domestic cats (*Felis catus*) in the northwest of Iran: Evidence for SARS-CoV-2 circulating between human and cats. Virus Res..

[7] Kuchipudi S.V., Surendran-Nair M., Ruden R.M., Yon M., Nissly R.H., Vandegrift K.J., Nelli R.K., Li L., Jayarao B.M., Maranas C.D. (2022). Multiple spillovers from humans and onward transmission of SARS-CoV-2 in white-tailed deer. Proc. Natl. Acad. Sci. USA.

[8] Hale V.L., Dennis P.M., McBride D.S., Nolting J.M., Madden C., Huey D., Ehrlich M., Grieser J., Winston J., Lombardi D. (2021). SARS-CoV-2 infection in free-ranging white-tailed deer. Nature.

[9] McAloose D., Laverack M., Wang L., Killian M.L., Caserta L.C., Yuan F., Mitchell P.K., Queen K., Mauldin M.R., Cronk B.D. (2020). From People to Panthera: Natural SARS-CoV-2 Infection in Tigers and Lions at the Bronx Zoo. MBio.

[10] Mishra A., Kumar N., Bhatia S., Aasdev A., Kanniappan S., Sekhar A.T., Gopinadhan A., Silambarasan R., Sreekumar C., Dubey C.K. (2021). SARS-CoV-2 Delta Variant among Asiatic Lions, India. Emerg. Infect. Dis..

[11] Koeppel K.N., Mendes A., Strydom A., Rotherham L., Mulumba M., Venter M. (2022). SARS-CoV-2 Reverse Zoonoses to Pumas and Lions, South Africa. Viruses.

[12] Memish Z.A., Mishra N., Olival K.J., Fagbo S.F., Kapoor V., Epstein J.H., Alhakeem R., Durosinloun A., Al Asmari M., Islam A. (2013). Middle East respiratory syndrome coronavirus in bats, Saudi Arabia. Emerg. Infect. Dis..

[13] Brook C.E., Dobson A.P. (2015). Bats as ‘special’ reservoirs for emerging zoonotic pathogens. Trends Microbiol..

[14] Wong S., Lau S., Woo P., Yuen K.-Y. (2007). Bats as a continuing source of emerging infections in humans. Rev. Med. Virol..

[15] Olival K.J., Cryan P.M., Amman B.R., Baric R.S., Blehert D.S., Brook C.E., Calisher C.H., Castle K.T., Coleman J.T.H., Daszak P. (2020). Possibility for reverse zoonotic transmission of SARS-CoV-2 to free-ranging wildlife: A case study of bats. PLoS Pathog..

[16] Latinne A., Hu B., Olival K.J., Zhu G., Zhang L., Li H., Chmura A.A., Field H.E., Zambrana-Torrelio C., Epstein J.H. (2020). Origin and cross-species transmission of bat coronaviruses in China. Nat. Commun..

[17] Ge X.-Y., Li J.-L., Yang X.-L., Chmura A.A., Zhu G., Epstein J.H., Mazet J.K., Hu B., Zhang W., Peng C. (2013). Isolation and characterization of a bat SARS-like coronavirus that uses the ACE2 receptor. Nature.

[18] Corman V.M., Ithete N.L., Richards L.R., Schoeman M.C., Preiser W., Drosten C., Drexler J.F., Perlman S. (2014). Rooting the Phylogenetic Tree of Middle East Respiratory Syndrome Coronavirus by Characterization of a Conspecific Virus from an African Bat. J. Virol..

[19] Ithete N.L., Stoffberg S., Corman V.M., Cottontail V.M., Richards L.R., Schoeman M.C., Drosten C., Drexler J.F., Preiser W. (2013). Close relative of human Middle East respiratory syndrome coronavirus in bat, South Africa. Emerg. Infect. Dis..

[20] Yang Y., Du L., Liu C., Wang L., Ma C., Tang J., Baric R.S., Jiang S., Li F. (2014). Receptor usage and cell entry of bat coronavirus HKU4 provide insight into bat-to-human transmission of MERS coronavirus. Proc. Natl. Acad. Sci. USA.

[21] Huang Y.W., Dickerman A.W., Piñeyro P., Li L., Fang L., Kiehne R., Opriessnig T., Meng X.J. (2013). Origin, evolution, and genotyping of emergent porcine epidemic diarrhea virus strains in the United States. mBio.

[22] Schorr R.A., Siemers J.L. (2021). Population dynamics of little brown bats (*Myotis lucifugus*) at summer roosts: Apparent survival, fidelity, abundance, and the influence of winter conditions. Ecol. Evol..

[23] Subudhi S., Rapin N., Bollinger T.K., Hill J.E., Donaldson M.E., Davy C.M., Warnecke L., Turner J.M., Kyle C.J., Willis C.K.R. (2017). A persistently infecting coronavirus in hibernating Myotis lucifugus, the North American little brown bat. J. Gen. Virol..

[24] Hall J.S., Knowles S., Nashold S.W., Ip H.S., Leon A.E., Rocke T., Keller S., Carossino M., Balasuriya U., Hofmeister E. (2021). Experimental challenge of a North American bat species, big brown bat (*Eptesicus fuscus*), with SARS-CoV-2. Transbound. Emerg. Dis..

[25] Schlottau K., Rissmann M., Graaf A., Schön J., Sehl J., Wylezich C., Höper D., Mettenleiter T.C., Balkema-Buschmann A., Harder T. (2020). SARS-CoV-2 in fruit bats, ferrets, pigs, and chickens: An experimental transmission study. Lancet Microbe.

[26] Hall J.S., Hofmeister E., Ip H.S., Nashold S.W., Leon A.E., Malavé C.M., Falendysz E.A., Rocke T.E., Carossino M., Balasuriya U. (2022). Experimental infection of Mexican free-tailed bats (*Tadarida brasiliensis*) with SARS-CoV-2. bioRxiv.

[27] Simmons N.B., Cirranello A.L. (2022). Bat Species of the World: A taxonomic and geographic database. Accessed.

[28] Reeder D.M., Field K.A., Slater M.H. (2016). Balancing the Costs of Wildlife Research with the Benefits of Understanding a Panzootic Disease, White-Nose Syndrome. ILAR J..

[29] Beyerstedt S., Casaro E.B., Rangel É.B. (2021). COVID-19: Angiotensin-converting enzyme 2 (ACE2) expression and tissue susceptibility to SARS-CoV-2 infection. Eur. J. Clin. Microbiol. Infect. Dis..

[30] Wu L., Chen Q., Liu K., Wang J., Han P., Zhang Y., Hu Y., Meng Y., Pan X., Qiao C. (2020). Broad host range of SARS-CoV-2 and the molecular basis for SARS-CoV-2 binding to cat ACE2. Cell Discov..

[31] Chiocchetti R., Galiazzo G., Fracassi F., Giancola F., Pietra M. (2020). ACE2 Expression in the Cat and the Tiger Gastrointestinal Tracts. Front. Vet. Sci..

[32] Li L., Han P., Huang B., Xie Y., Li W., Zhang D., Han P., Xu Z., Bai B., Zhou J. (2022). Broader-species receptor binding and structural bases of Omicron SARS-CoV-2 to both mouse and palm-civet ACE2s. Cell Discov..

[33] Zhou J., Li C., Liu X., Chiu M.C., Zhao X., Wang D., Wei Y., Lee A., Zhang A.J., Chu H. (2020). Infection of bat and human intestinal organoids by SARS-CoV-2. Nat. Med..

[34] Crawford K.H.D., Eguia R., Dingens A.S., Loes A.N., Malone K.D., Wolf C.R., Chu H.Y., Tortorici M.A., Veesler D., Murphy M. (2020). Protocol and Reagents for Pseudotyping Lentiviral Particles with SARS-CoV-2 Spike Protein for Neutralization Assays. Viruses.

[35] Stadlbauer D., Amanat F., Chromikova V., Jiang K., Strohmeier S., Arunkumar G.A., Tan J., Bhavsar D., Capuano C., Kirkpatrick E. (2020). SARS-CoV-2 Seroconversion in Humans: A Detailed Protocol for a Serological Assay, Antigen Production, and Test Setup. Curr. Protoc. Microbiol..

[36] Gontu A., Marlin E.A., Ramasamy S., Neerukonda S., Anil G., Morgan J., Quraishi M., Chen C., Boorla V.S., Nissly R.H. (2022). Development and Validation of Indirect Enzyme-Linked Immunosorbent Assays for Detecting Antibodies to SARS-CoV-2 in Cattle, Swine, and Chicken. Viruses.

[37] Kuchipudi S.V., Nelli R., White G.A., Bain M., Chang K.C., Dunham S. (2009). Differences in influenza virus receptors in chickens and ducks: Implications for interspecies transmission. J. Mol. Genet. Med..

[38] Waterhouse A., Bertoni M., Bienert S., Studer G., Tauriello G., Gumienny R., Heer F.T., de Beer T.A.P., Rempfer C., Bordoli L. (2018). SWISS-MODEL: Homology modelling of protein structures and complexes. Nucleic Acids Res..

[39] Wang Q., Zhang Y., Wu L., Niu S., Song C., Zhang Z., Lu G., Qiao C., Hu Y., Yuen K.Y. (2020). Structural and Functional Basis of SARS-CoV-2 Entry by Using Human ACE2. Cell.

[40] Honorato R.V., Koukos P.I., Jiménez-García B., Tsaregorodtsev A., Verlato M., Giachetti A., Rosato A., Bonvin A.M.J.J. (2021). Structural Biology in the Clouds: The WeNMR-EOSC Ecosystem. Front. Mol. Biosci..

[41] Alford R.F., Leaver-Fay A., Jeliazkov J.R., O’Meara M.J., DiMaio F.P., Park H., Shapovalov M.V., Renfrew P.D., Mulligan V.K., Kappel K. (2017). The Rosetta All-Atom Energy Function for Macromolecular Modeling and Design. J. Chem. Theory Comput..

[42] Stranges P.B., Kuhlman B. (2013). A comparison of successful and failed protein interface designs highlights the challenges of designing buried hydrogen bonds. Protein Sci..

[43] Lyskov S., Gray J.J. (2008). The RosettaDock server for local protein–protein docking. Nucleic Acids Res..

[44] Madhavi Sastry G., Adzhigirey M., Day T., Annabhimoju R., Sherman W. (2013). Protein and ligand preparation: Parameters, protocols, and influence on virtual screening enrichments. J. Comput.-Aided Mol. Des..

[45] Roos K., Wu C., Damm W., Reboul M., Stevenson J.M., Lu C., Dahlgren M.K., Mondal S., Chen W., Wang L. (2019). OPLS3e: Extending Force Field Coverage for Drug-Like Small Molecules. J. Chem. Theory Comput..

[46] Jorgensen W.L., Chandrasekhar J., Madura J.D., Impey R.W., Klein M.L. (1983). Comparison of simple potential functions for simulating liquid water. J. Chem. Phys..

[47] Bowers K.J., Chow D.E., Xu H., Dror R.O., Eastwood M.P., Gregersen B.A., Klepeis J.L., Kolossvary I., Moraes M.A., Sacerdoti F.D. Scalable Algorithms for Molecular Dynamics Simulations on Commodity Clusters. Proceedings of the 2006 ACM/IEEE Conference on Supercomputing.

[48] Darden T., York D., Pedersen L. (1993). Particle mesh Ewald: An N⋅log(N) method for Ewald sums in large systems. J. Chem. Phys..

[49] Kräutler V., van Gunsteren W.F., Hünenberger P.H. (2001). A fast SHAKE algorithm to solve distance constraint equations for small molecules in molecular dynamics simulations. J. Comput. Chem..

[50] Hamming I., Timens W., Bulthuis M.L.C., Lely A.T., Navis G.J., van Goor H. (2004). Tissue distribution of ACE2 protein, the functional receptor for SARS coronavirus. A first step in understanding SARS pathogenesis. J. Pathol..

[51] Li M.-Y., Li L., Zhang Y., Wang X.-S. (2020). Expression of the SARS-CoV-2 cell receptor gene ACE2 in a wide variety of human tissues. Infect. Dis. Poverty.

[52] Li R., Qiao S., Zhang G. (2020). Analysis of angiotensin-converting enzyme 2 (ACE2) from different species sheds some light on cross-species receptor usage of a novel coronavirus 2019-nCoV. J. Infect..

[53] Zang R., Castro M.F.G., McCune B.T., Zeng Q., Rothlauf P.W., Sonnek N.M., Liu Z., Brulois K.F., Wang X., Greenberg H.B. (2020). TMPRSS2 and TMPRSS4 promote SARS-CoV-2 infection of human small intestinal enterocytes. Sci. Immunol..

[54] Hoffmann M., Kleine-Weber H., Schroeder S., Krüger N., Herrler T., Erichsen S., Schiergens T.S., Herrler G., Wu N.-H., Nitsche A. (2020). SARS-CoV-2 Cell Entry Depends on ACE2 and TMPRSS2 and Is Blocked by a Clinically Proven Protease Inhibitor. Cell.

[55] Marze N.A., Roy Burman S.S., Sheffler W., Gray J.J. (2018). Efficient flexible backbone protein–protein docking for challenging targets. Bioinformatics.

[56] Schreiber G., Haran G., Zhou H.X. (2009). Fundamental Aspects of Protein−Protein Association Kinetics. Chem. Rev..

[57] Delaune D., Hul V., Karlsson E.A., Hassanin A., Ou T.P., Baidaliuk A., Gámbaro F., Prot M., Tu V.T., Chea S. (2021). A novel SARS-CoV-2 related coronavirus in bats from Cambodia. Nat. Commun..

[58] Daly J.L., Simonetti B., Klein K., Chen K.E., Williamson M.K., Antón-Plágaro C., Shoemark D.K., Simón-Gracia L., Bauer M., Hollandi R. (2020). Neuropilin-1 is a host factor for SARS-CoV-2 infection. Science.

[59] Wang K., Chen W., Zhou Y.-S., Lian J.-Q., Zhang Z., Du P., Gong L., Zhang Y., Cui H.-Y., Geng J.-J. (2020). SARS-CoV-2 invades host cells via a novel route: CD147-spike protein. bioRxiv.

[60] Hoffmann M., Kleine-Weber H., Pöhlmann S. (2020). A Multibasic Cleavage Site in the Spike Protein of SARS-CoV-2 Is Essential for Infection of Human Lung Cells. Mol. Cell.

[61] Yan H., Jiao H., Liu Q., Zhang Z., Xiong Q., Wang B.-J., Wang X., Guo M., Wang L.-F., Lan K. (2021). ACE2 receptor usage reveals variation in susceptibility to SARS-CoV and SARS-CoV-2 infection among bat species. Nat. Ecol. Evol..

[62] Shi J., Wen Z., Zhong G., Yang H., Wang C., Huang B., Liu R., He X., Shuai L., Sun Z. (2020). Susceptibility of ferrets, cats, dogs, and other domesticated animals to SARS-coronavirus 2. Science.

[63] Ma B., Zhang Z., Li Y., Lin X., Gu N. (2022). Evaluation of Interactions between SARS-CoV-2 RBD and Full-Length ACE2 with Coarse-Grained Molecular Dynamics Simulations. J. Chem. Inf. Model..

[64] Yoshida N., Maruyama Y., Mitsutake A., Kuroda A., Fujiki R., Kanemaru K., Okamoto D., Kobryn A.E., Gusarov S., Nakano H. (2022). Computational Analysis of the SARS-CoV-2 RBD-ACE2-Binding Process Based on MD and the 3D-RISM Theory. J. Chem. Inf. Model..

[65] Huang X., Zhang C., Pearce R., Omenn G.S., Zhang Y. (2020). Identifying the zoonotic origin of SARS-CoV-2 by modeling the binding affinity between the spike receptor-binding domain and host ACE2. J. Proteome Res..

[66] Alexander M.R., Schoeder C.T., Brown J.A., Smart C.D., Moth C., Wikswo J.P., Capra J.A., Meiler J., Chen W., Madhur M.S. (2020). Predicting susceptibility to SARS-CoV-2 infection based on structural differences in ACE2 across species. FASEB J..

[67] Elaswad A., Fawzy M., Basiouni S., Shehata A.A. (2020). Mutational spectra of SARS-CoV-2 isolated from animals. PeerJ.

[68] Harcourt J., Tamin A., Lu X., Kamili S., Sakthivel S.K., Murray J., Queen K., Tao Y., Paden C.R., Zhang J. (2020). Severe Acute Respiratory Syndrome Coronavirus 2 from Patient with Coronavirus Disease, United States. Emerg. Infect. Dis..

[69] Damas J., Hughes G.M., Keough K.C., Painter C.A., Persky N.S., Corbo M., Hiller M., Koepfli K.-P., Pfenning A.R., Zhao H. (2020). Broad host range of SARS-CoV-2 predicted by comparative and structural analysis of ACE2 in vertebrates. Proc. Natl. Acad. Sci. USA.

[70] Zhang W., Shi K., Geng Q., Ye G., Aihara H., Li F. (2022). Structural basis for mouse receptor recognition by SARS-CoV-2 omicron variant. Proc. Natl. Acad. Sci. USA.

[71] Frances-Monerris A., Hognon C., Miclot T., Garcia-Iriepa C., Iriepa I., Terenzi A., Grandemange S., Barone G., Marazzi M., Monari A. (2020). Molecular basis of SARS-CoV-2 infection and rational design of potential antiviral agents: Modeling and simulation approaches. J. Proteome Res..

[72] Gonzalez T.R., Martin K.P., Barnes J.E., Patel J.S., Ytreberg F.M. (2020). Assessment of software methods for estimating protein-protein relative binding affinities. PLoS ONE.

[73] Chen C., Boorla V.S., Banerjee D., Chowdhury R., Cavener V.S., Nissly R.H., Gontu A., Boyle N.R., Vandegrift K., Nair M.S. (2021). Computational prediction of the effect of amino acid changes on the binding affinity between SARS-CoV-2 spike RBD and human ACE2. Proc. Natl. Acad. Sci. USA.

[74] Liu X., Luo Y., Li P., Song S., Peng J. (2021). Deep geometric representations for modeling effects of mutations on protein-protein binding affinity. PLoS Comput. Biol..

[75] Greaney A.J., Loes A.N., Crawford K.H., Starr T.N., Malone K.D., Chu H.Y., Bloom J.D. (2021). Comprehensive mapping of mutations in the SARS-CoV-2 receptor-binding domain that affect recognition by polyclonal human plasma antibodies. Cell Host Microbe.

