# Supplementary material for: Little Brown Bats (Myotis lucifugus) Support the Binding of SARS-CoV-2 Spike and Are Likely Susceptible to SARS-CoV-2 Infection
Source: Viruses. 2023 Apr 30;15(5):1103. doi: 10.3390/v15051103 (PMC10223257; doi:10.3390/v15051103)
Supplement: Supplementary file 1 [file viruses-15-01103-s001.zip › viruses-2355047-supplementary.pdf]

Supplementary Material

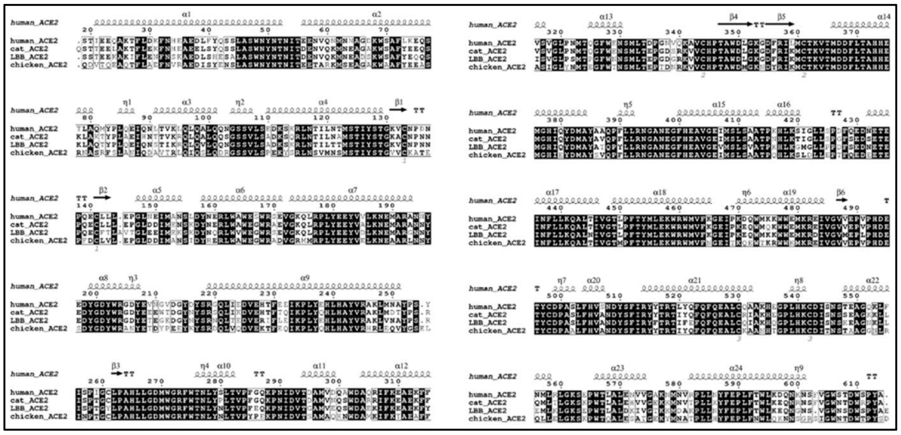

Figure S1. Sequence alignment of ACE2 proteins of human, cat, LBB, and chicken. The sequence numbering follows the crystal structure 6LZG of human ACE2-RBD complex. The top row shows the secondary structure assignments of human ACE2 protein as per the 6LZG crystal structure. Fully conserved positions among the sequences are highlighted in black with white text, while conserved positions with similar amino-acid substitutions are outlined with a black box and bold black text, and the rest of the positions are not conserved.

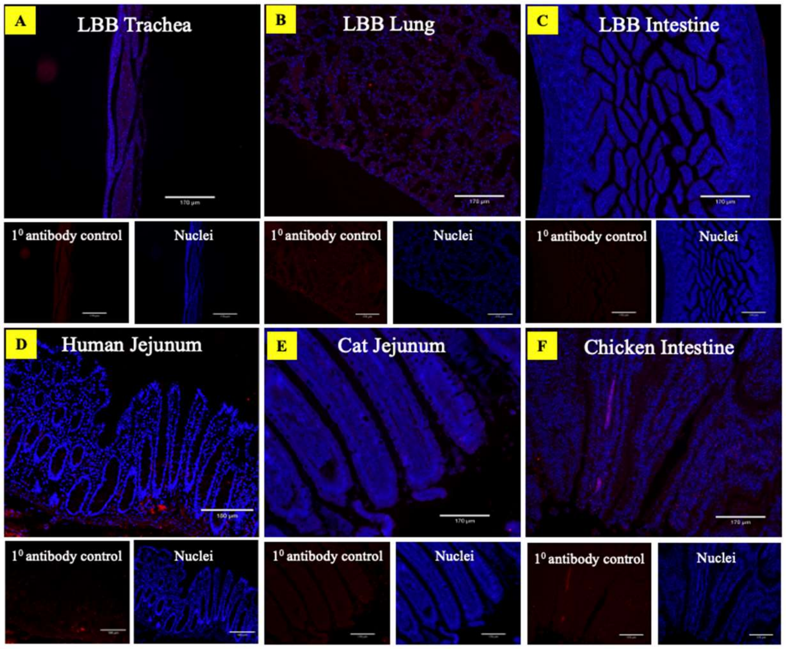

Figure S2: ACE2 primary antibody mock staining. The LBB trachea (A), lung (B), intestine (C), human jejunum (D), cat jejunum (E), and chicken intestine (F) were mock stained for ACE2 protein expression by omitting the ACE2 primary antibody. All the tissues were stained with the secondary Anti-Rabbit IgG H&L antibody (Alexa Fluor 647) to help determine the non-specific background staining caused by the secondary antibody and with DAPI nuclear stain (blue).

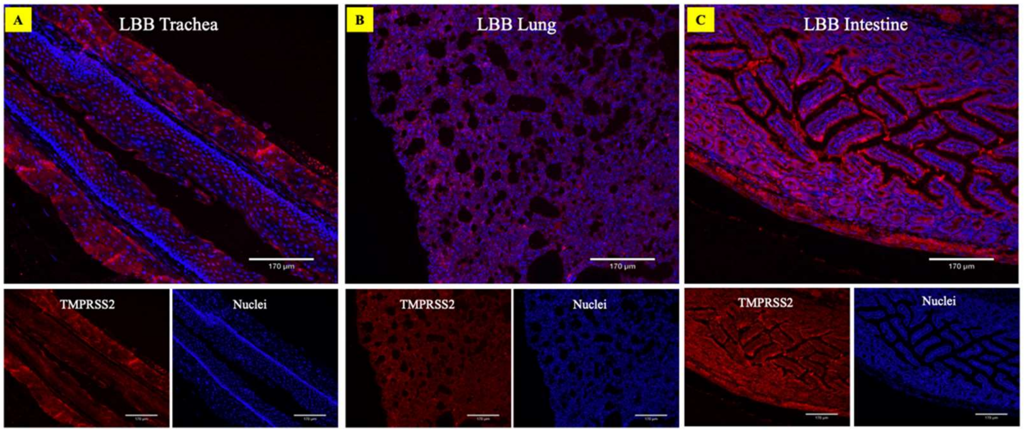

Figure S3. Expression of TMPRSS2 protein receptors in the trachea and intestine of the little brown bat. Composite fluorescent images show expression of TMPRSS2 protein receptor (red) on the (A) trachea, (B) lung, and (C) intestine. The receptor was predominantly expressed on the mucosal epithelium of the trachea (A), whereas it was moderately expressed in the lung tissue (B). The epithelial lining of the intestinal villi (C) showed a high TMPRSS2 expression. The tissue sections were stained with rabbit polyclonal antibodies to TMPRSS2 (red) and DAPI nuclear stain (blue).

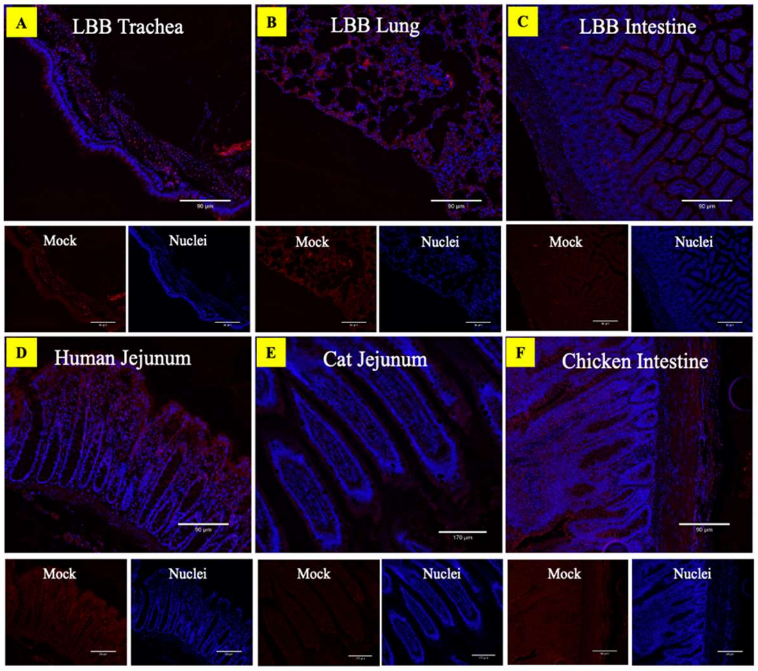

Figure S4: Mock staining for ps-SARS-CoV-2 virus binding assay. The LBB trachea (A), lung (B), intestine (C), human jejunum (D), cat jejunum (E), and chicken intestine (F) were mock stained for ps-SARS-CoV-2 virus binding assay by using TBS instead of the virus. The tissues were further stained with primary anti-SARS-CoV-2 spike protein S1 monoclonal antibody followed by staining with secondary Anti-Rabbit IgG H&L antibody (red). Staining with the primary and secondary antibodies helped determine the level of non-specific background fluorescence caused by the staining. The tissue slides were mounted using Prolong gold antifade mountant with nuclear stain, DAPI (blue).

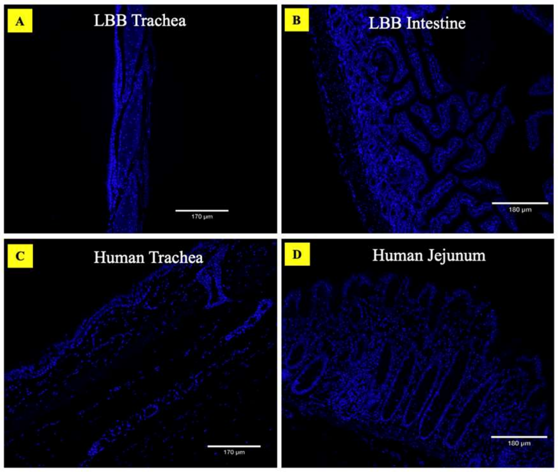

Figure S5: Mock staining for recombinant SARS-CoV-2/RBD antigen-binding assay. The LBB trachea (A), intestine (B), human trachea (C), and human jejunum (D) were mock stained for SARS-CoV-2/RBD antigen, using TBS instead of the anti-his antibody. No secondary antibody was used in this assay. The tissue slides were mounted using Prolong gold antifade mountant with nuclear stain, DAPI (blue).

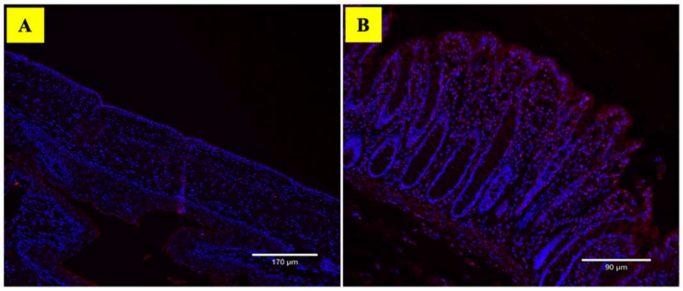

Figure S6: Mock staining of human tissues for SARS-CoV-2 spike pseudovirus binding assay. The human trachea (A) and jejunum (B) were mock stained for SARS-CoV-2 spike pseudovirus S1, after incubation with cell culture media as control. The tissues were stained with a secondary Anti-Rabbit IgG H&L antibody (red) and mounted using Prolong gold antifade mountant with nuclear stain, DAPI (blue). The secondary antibody showed minimal background.

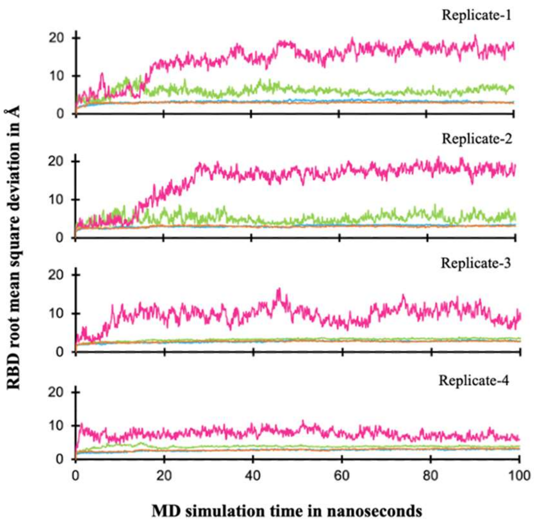

Figure S7. Molecular dynamics simulation trajectories showing RBD deviation during four independent 100ns replicate simulations.

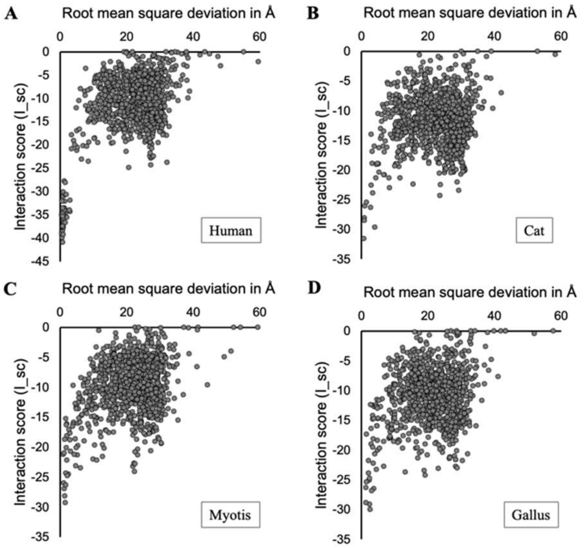

Figure S8. Docking funnels for Rosetta docking simulations for ACE2-RBD complexes. (A) Human ACE2-RBD taken from the crystal structure PDB-id: 6LZG produces a steep funnel with 41 high-confidence models. (B) Cat ACE2-RBD taken from the crystal structure PDB-id: 7C8D produces a narrower funnel with 12 high-confidence models. (C) Myotis ACE2-RBD generated from HADDOCK server produces a funnel with 17 high-confidence models. (D) Gallus ACE2-RBD generated from HADDOCK server produces a folding funnel with 19 high-confidence models.

Table S1: Species of interest and ACE2 GenBank identification numbers used in phylogenetic analyses.

Species common name	Species scientific name	GenBank ID
Human	*Homo sapiens*	NP_001358344
Cat	*Felis catus*	NP_001034545
Chicken	*Gallus gallus*	XP_416822
Little Brown Bat	*Myotis lucifugus*	XP_023609437
Big Brown Bat	*Eptesicus fuscus*	XP_008153150
Mexican free-tailed bat	*Tadarida brasiliensis*	QLF98520
